# *Edwardsiella piscicida* YefM-YoeB: A Type II Toxin-Antitoxin System That Is Related to Antibiotic Resistance, Biofilm Formation, Serum Survival, and Host Infection

**DOI:** 10.3389/fmicb.2021.646299

**Published:** 2021-03-01

**Authors:** Dongmei Ma, Hanjie Gu, Yanjie Shi, Huiqin Huang, Dongmei Sun, Yonghua Hu

**Affiliations:** ^1^College of Life Science and Technology, Heilongjiang Bayi Agricultural University, Daqing, China; ^2^Institute of Tropical Bioscience and Biotechnology, Hainan Academy of Tropical Agricultural Resource, CATAS, Haikou, China; ^3^Laboratory for Marine Biology and Biotechnology, Pilot National Laboratory for Marine Science and Technology, Qingdao, China; ^4^Hainan Provincial Key Laboratory for Functional Components Research and Utilization of Marine Bio-resources, Haikou, China

**Keywords:** *Edwardsiella piscicida*, toxin-antitoxin, YefM-YoeB, adversity, virulence

## Abstract

The emergence of drug resistant bacteria is a tricky and confronted problem in modern medicine, and one of important reasons is the widespread of toxin-antitoxin (TA) systems in pathogenic bacteria. *Edwardsiella piscicida* (also known as *E. tarda*) is the leading pathogen threatening worldwide fresh and seawater aquaculture industries and has been considered as a model organism for studying intracellular and systemic infections. However, the role of type II TA systems are completely unknown in aquatic pathogenic bacteria. In this study, we identified and characterized a type II TA system, YefM-YoeB, of *E. piscicida*, where YefM is the antitoxin and YoeB is the toxin. *yefM* and *yoeB* are co-expressed in a bicistronic operon. When expressed in *E. coli*, YoeB cause bacterial growth arrest, which was restored by the addition of YefM. To investigate the biological role of the TA system, two markerless *yoeB* and *yefM-yoeB* in-frame mutant strains, TX01Δ*yoeB* and TX01Δ*yefM-yoeB*, were constructed, respectively. Compared to the wild strain TX01, TX01Δ*yefM-yoeB* exhibited markedly reduced resistance against oxidative stress and antibiotic, and markedly reduced ability to form persistent bacteria. The deletion of *yefM-yoeB* enhanced the bacterial ability of high temperature tolerance, biofilm formation, and host serum resistance, which is the first study about the relationship between type II TA system and serum resistance. *In vitro* infection experiment showed that the inactivation of *yefM-yoeB* greatly enhanced bacterial capability of adhesion in host cells. Consistently, *in vivo* experiment suggested that the *yefM-yoeB* mutation had an obvious positive effect on bacteria dissemination of fish tissues and general virulence. Introduction of a trans-expressed *yefM-yoeB* restored the virulence of TX01Δ*yefM-yoeB*. These findings suggest that YefM-YoeB is involved in responding adverse circumstance and pathogenicity of *E. piscicida*. In addition, we found that YefM-YoeB negatively autoregulated the expression of *yefM-yoeB* and YefM could directly bind with own promoter. This study provides first insights into the biological activity of type II TA system YefM-YoeB in aquatic pathogenic bacteria and contributes to understand the pathogenesis of *E. piscicida*.

## Introduction

*Edwardsiella* was isolated from infected humans and animals and identified as a new genus of Enterobacteriaceae in 1965 (Ewing et al., 1965). The *Edwardsiella* genus was classified into five species, including *E. piscicida*, *E. anguillarum*, *E. ictaluri, E. tarda*, and *E. hoshinae* (Abayneh et al., 2013; Leung et al., 2019). The front three species belong to fish pathogens. *E. piscicida* (formerly included in *E. tarda*) is recognized as one of the most severe pathogens in cultivating fishery (Leung et al., 2012). It can infect not only freshwater and marine fish at the same time, causing a large number of infections and deaths of fish, but also a zoonotic pathogen with a wide host range, including mammals and reptiles (Janda and Abbott, 1993; Mohanty and Sahoo, 2007; Park et al., 2012). In recent years, researches on the pathogenic mechanism of *E. piscicida* have become a research hotspot in the field of aquatic animal diseases. A variety of pathogenic factors and systems, such as adhesion factors, iron uptake regulators, antiserum ability, type III and VI secretion systems, hemolysin, catalase, cytotoxin, TAM, two-component system, density induction systems, flagellin, lysozyme inhibitors, intracellular survival mechanisms, and some environmental stress factors have been discovered and studied (Yin et al., 2018; Leung et al., 2019; He et al., 2020; Li et al., 2020; Yoon et al., 2020). However, toxin-antitoxin (TA) system, an important stress and antibiotic resistance factor/system, are totally unknown in *E. piscicida*.

The rapid rise in the emergence of drug-resistant bacteria is a tricky and confronted problem in modern medicine. One of important reasons for emergence of antibiotic-resistant bacteria is the widespread of TA systems in bacteria (Równicki et al., 2020). The first identified toxin-antitoxin (TA) systems were characterized as “plasmid addiction modules” (Ogura and Hiraga, 1983; Guo et al., 2019). Subsequently, TA systems were discovered to be extensive as genetic elements in bacteria and archaea, including chromosomally encoded TA systems. In the past 30 years, according to the nature of the antitoxin and the composition of the TA system, a total of six types of TA (Type I–Type VI) have been discovered (Page and Peti, 2016). Amongst these TA systems, type II TA systems are the most prevalent and most extensively studied (Równicki et al., 2020). They are usually composed of two co-transcribed genes, encoding stable toxin proteins and easily degradable antitoxin proteins. In normal growing cells, the antitoxin combines with its homologous toxin to produce a harmless protein-protein complex that prevents the toxin from exerting its toxicity (Yamaguchi et al., 2011). The antitoxin binds to the upstream of the TA operon to inhibit the expression of the operon, and the toxin acts as an auxiliary inhibitor combined with the antitoxin to strengthen this inhibition, so the level of intracellular TA complex is regulated by the antitoxin and TA complex together (Gerdes and Maisonneuve, 2012). Under certain stress conditions, the antitoxins are degraded by proteases to release stable toxin proteins, at the same time, the inhibition of the operon is lifted, resulting in the accumulation of toxins and the activation of toxic effects (Brzozowska and Zielenkiewicz, 2013; Schuster and Bertram, 2013).

Persisters are rare bacteria in a bacterial population that could resist to lethal antibiotics and that could account for the relapse of infections. Environmental stresses and growth phases influence the formation of persister cells (Bernier et al., 2013; Helaine et al., 2014). An important reason for the formation of persister cells is TA systems (Verstraeten et al., 2015). Currently, a number of type II TA systems have been identified, and they are widely present in all kinds of pathogens, such as *E. coli*, *Streptococcus pneumoniae*, *Pseudomonas aeruginosa*, and *Staphylococcus aureus* (Lobato-Márquez et al., 2016; Wood and Wood, 2016; Chan et al., 2018; Wen et al., 2018). Reports have shown that type II toxin-antitoxin systems can ensure the safety and stability of mobile genetic elements, induce the formation of persistent cells, participate in biofilm formation, stress response, and pathogenicity (Wang and Wood, 2011; Harms et al., 2016; Oron-Gottesman et al., 2016; Sun et al., 2017; Ma et al., 2019). Recently, an increasing number of studies are devoted to the role of type II TA systems in pathogenicity (Równicki et al., 2020).

Among type II TA systems, YefM-YoeB is one of most frequently encountered TA systems in many pathogenic bacteria. In *S. aureus*, there are two distinct oligomeric assemblies: heterotetramer (YoeB-YefM2-YoeB) and heterohexamer (YoeB-YefM2-YefM2-YoeB) (Xue et al., 2020). In *S. pneumoniae*, it is reported that YefM-YoeB participates in oxidative stress and biofilm formation (Chan et al., 2018). In *E. coli*, YoeB toxin is activated during thermal stress (Janssen et al., 2015) and YefM-YoeB is involved in the niche-specific colonization and stress resistance (Norton and Mulvey, 2012). Currently, there is no report about the type II TA systems in aquatic pathogenic bacteria and their roles are totally unknown. In this study, we for the first time identified and characterized a type II TA system, YefM-YoeB, in *E. piscicida*. We investigated the role of YefM-YoeB in adversity adaptation and pathogenicity. Our results suggested that the whole TA system YefM-YoeB was related to antibiotic resistance, persistence bacteria formation, oxidative resistance, biofilm formation, host serum resistance, and host infection. Antitoxin YefM negatively autoregulated its own expression and toxin YoeB remarkably enhance the regulatory effect of YefM. This study provides first insights into the biological activity of type II TA system in aquatic pathogenic bacteria and will help us understand the pathogenic mechanism.

## Materials and Methods

### Bacteria and Growth Conditions

*E. coli* BL21 (DE3) was purchased from TransGen (Beijing, China). *E. coli* S17-1λpir was purchased from Biomedal (Seville, Spain). *E. piscicida* TX01 was isolated from diseased fish. Bacteria were cultured in Luria-Bertani broth (LB) at 37°C (for *E. coli*) or 28°C (for *E. piscicida*). Where indicated, Ampicillin, Kanamycin, chloramphenicol, tetracycline, and polymyxin B, were supplemented at the concentration of 100, 50, 30, 15, and 100 μg/mL (Fang et al., 2019).

### Total RNA Isolation, cDNA Generation, and Co-transcription Analysis

Overnight cultures of *E. piscicida* were diluted 1:100 in LB and grown at 28°C to exponential phase. The genomic DNA and total RNA of bacteria were isolated with TIANamp Bacteria DNA Kit (TIANGEN, Beijing, China) and HP Total RNA kit (Omega Bio-Tek, United States). Total RNA was used to synthesize the cDNA with random primers and PrimeScript Reverse Transcriptase (Thermo Fisher Scientific, Wilmington, DE, United States). cDNA was used as template for PCR amplification, simultaneously, genomic DNA as a positive control and total RNA as a negative control for the PCR. The PCR were analyzed with the primers YoeBRTF/YoeBRTR for *yoeB* (control) and YefM-YoeBRTF/YefM-YoeBRTR for *yefM-yoeB*. Primers YefM-YoeBRTF/YefM-YoeBRTR were annealed to the 5′ end of *yefM* and 3′ end of *YoeB* coding region, respectively, and Gel electrophoresis was analyzed the PCR products amplified with genomic DNA, cDNA, and RNA. The primers used in this study were listed in Table 1.

**TABLE 1:** Primers used in this study.

**Primer**	**Sequence (5′–3′)**
YoeBKOF1	GGATCCCGGTACGTCAGTTTTATCAAT(BamHI)
YoeBKOR1	CGGTGCTGGCCGTTTAGGGTAAAATTAATAG
YoeBKOF2	TAAACGGCCAGCACCGCCTCGTTTACAGT
YoeBKOR2	GGATCCGGGAGATGCCGAGCTTTCTG(BamHI)
YoeBKOF3	GTTCATGTCTTGTACGCGAAT
YoeBKOR3	CCACCGTAACCGTGTTACCT
YefM-YoeBKOF1	GGATCCAGAGGAGGTGATGCTGG(BamHI)
YefM-YoeBKOR1	CAGGCAACGTTGGTTACACGGTTC
YefM-YoeBKOF2	TAACCAACGTTGCCTGCCGCTTTC
YefM-YoeBKOR2	GGATCCCTGGGAATCCATGAGC(BamHI)
YefM-YoeBKOF3	TGTTACCTTCTCGGATGCC
YefM-YoeBKOR3	CAAGAGGGCCGGGATT
YefM-YoeBC-F	GATATCGAGGAGGTGATGCTGG(EcoR V)
YefM-YoeBC-R	GATATCTCATTTATCCCCGTAGTGAAAG(EcoR V)
YefM-YoeBP-F	GATATCGAGGAGGTGATGCTGG(EcoR v)
YefM-YoeBP-R	GATATCTCGCATCCTTAAGAGTGGT(EcoR V)
YefMPro-F	GAATTCATGCACACTGTTACCTTCTC(EcoRI)
YefMPro-F	CTCGAGATAGTCCACTGTGACAACTTCG(XhoI)
YefMF4	GGATCCATGCACACTGTTACCTTCTC(BamHI)
YefMR4	CATATGATAGTCCACTGTGACAACTTCG(NdeI)
YoeBF4	GGATCCTTGTCACAGTGGACTATTAATT(BamHI)
YoeBR4	CATATGTCATTTATCCCCGTAGTGAAAG(NdeI)
YoeBRTF	ATGTCACAGTGGACTATTAATTTTAC
YoeBRTR	TTATTTATCCCCGTAGTGAAAGC
YefM-YoeBRTF	GATACCATGAACCGTGTAACCAACA
YefM-YoeBRTR	TAGTGAAAGCGGCAGGCAAC

### Endogenous Toxicity of YoeB Assay

The endogenous toxicity of the toxin YoeB was determined by growth in liquid and solid medium. To construct the overexpression plasmid pTYefM and pETYoeB, ORFs of *yefM* and *yoeB* were amplified with the primer pairs YefMF4/YefMR4 and YoeBF4/YoeBR4, respectively. The amplified fragments were inserted into the pBT3 (Zhang et al., 2008) and pET28a, resulting in pTYefM and pETYoeB, which express YefM and YoeB, respectively. pTYefM, pETYoeB, and pET were transformed into *E. coli* BL21 (DE3), which resulted in BL21/pETYoeB, BL21/pTYefM+pETYoeB, and BL21/pTYefM+pET. These strains were cultured in LB with or without 0.5 mM IPTG and bacterial growth curve was monitored for 12 h (Wen et al., 2018). Bacteria was cultured to the exponential phase, diluted serially and dripped onto the LB plate with or without 0.5 mM IPTG, then the plates were cultured at 28°C for 24 h. The experiment was performed three times.

### Construction of yoeB and yefM-yoeB Mutation and yefM-yoeB Complementation

The primers used in this study were listed in Table 1. The construction of mutants was performed as reported as previously (Hu et al., 2014). To construct the *yoeB* knockout strain, TX01Δ*yoeB*, in-frame deletion of a 162 bp segment (residues 37–198) of *yoeB* was performed by overlap extension PCR as follows: the first overlap PCR was performed with the primer pair YoeBKOF1/R1, the second overlap PCR was performed with the primer pair YoeBKOF2/R2, and the fusion PCR was performed with the primer pair YoeBKOF1/R2. The PCR products amplified by primer pair YoeBKOF1/R2 were inserted into the suicide plasmid pDM4 at the BglII site, resulting in pDMYoeB. S17-1λpir was transformed with pDMYoeB and the transformants were conjugated with TX01 as described previously (Hu et al., 2014). The transconjugants were selected on LB agar plates supplemented with 10% sucrose. Colonies that grew up on LB plates with sucrose and was sensitive to chloramphenicol were analyzed by PCR with YoeBKOF3/YoeBKOR3. To confirm the in-frame deletion, the PCR products were subjected to DNA sequencing. To construct *yefM-yoeB* knockout strain, TX01Δ*yefM-yoeB*, deletion of a 403 bp segment (residues 67–469) of *yefM-yoeB* was performed by overlap PCR. The experiment was performed as described above. To constructed the complementary strain TX01Δ*yefM-yoeB*C, *yefM-yoeB* was amplified by PCR with primers YefM-YoeBC-F/R, was inserted into the plasmid pJR21 at the PmeI site, resulting in pJRYefM-YoeB. Then pJRYefM-YoeB was transformed into TX01Δ*yefM-yoeB* by conjugation. The complementary strain was named TX01Δ*yefM-yoeB*C.

### Resistance to Environmental Stress and to Non-immune Fish Serum

For high temperature resistance, TX01, TX01Δ*yoeB*, TX01Δ*yefM-yoeB*, and TX01Δ*yefM-yoeB*C were cultured in LB medium to exponential phase, then bacteria were transferred to fresh LB medium and cultured at 28 and 40°C. Bacterial cell density was measured at different time points by determining absorbance at OD_600_ as previously described (Fang et al., 2019).

For oxidative stress assay, the TX01, TX01Δ*yoeB*, TX01Δ*yefM-yoeB*, and TX01Δ*yefM-yoeB*C in exponential phase were washed with PBS and resuspended in PBS. Approximately 10^5^ bacterial cells were mixed with 250 μL 3.2 mM H_2_O_2_ or PBS (control). After incubating at 28°C for 60 min, the mixtures were serially diluted and plated in triplicate on LB agar plates. The plates were incubated at 28°C for 24 h, and the colonies that appeared on the plates were enumerated. The survival rate was calculated as follows: [(number of H_2_O_2_^–^treated cells)/(number of control cells)] × 100% (Wang et al., 2019). The experiment was performed three times.

To determine the non-immune fish serum tolerance, bacteria in exponential phase was washed with PBS three times. Ten microliters of bacteria (including approximately 10^5^ bacterial cells) were incubated with 50 μL non-immune fish serum or PBS (control) for 60 min. The mixtures were serially diluted and plated in triplicate on LB agar plates. The plates were incubated at 28°C for 24 h, and the colonies that appeared on the plates were enumerated. The survival rate was calculated as follows: [(number of serum-treated cells)/(number of control cells)] × 100% (Wang et al., 2019). The experiment was performed three times.

### Biofilm Assay

TX01, TX01Δ*yoeB*, TX01Δ*yefM-yoeB*, and TX01Δ*yefM-yoeB*C were cultured in LB medium to exponential phase and diluted to 10^5^ CFU/mL. Crystal violet staining of biofilm assay was performed as previously (Shi et al., 2019).

The observation of biofilms by confocal laser scanning microscopy (CLSM) was performed as described by Chan et al. (2018) and Shi et al. (2019). Briefly, TX01, TX01Δ*yoeB*, TX01Δ*yefM-yoeB*, and TX01Δ*yefM-yoeB*C were grown in LB medium on glass-bottom dishes for 24 h at 28°C. The dishes were rinsed to remove non-adherent bacteria and then stained with a LIVE/DEAD BacLight bacterial viability kit L-13152 (Invitrogen-Molecular Probes, Carlsbad, CA, United States) for observation of biofilms. The staining procedure involved incubation for 20 min at room temperature in the dark. The biofilms were observed using a Leica TCS-SP2-AOBS-UV confocal laser scanning microscope equipped with an argon ion laser. The experimental procedures were performed three times (Shi et al., 2019).

### Drug Sensitivity Test

The antibiotic drug susceptibility test was carried out in accordance with the operating standards of the paper diffusion method (Kirby-Bauer, K-B) recommended by the Clinical Laboratory Standards Institute (CLSI). TX01, TX01Δ*yoeB*, and TX01Δ*yefM-yoeB* were cultivated to OD_600_ = 0.5 (log phase), prepared a bacterial suspension (10^8^ CFU/mL) and plated it evenly on the MH solid plates. Thirty kinds of drug sensitive tablets (HANGWEI,Hangzhou, China) were attached to the plates, and three types of them were placed on each plate. After that, the plates were cultivated at 28°C for 24 h. Finally, the diameters of the inhibition zone on the plates were measured. The experiment was performed three times.

### Persister Cell Formation Assay

In order to study whether *yefM-yoeB* has an effect on the persister formation, TX01, TX01Δ*yoeB*, and TX01Δ*yefM-yoeB* were cultured overnight and the culture solution was diluted with 1:100 in LB. Bacterial culture was incubated at 28°C on a shaker at 200 rpm until the absorbance at 600 nm reached 0.5 (logarithmic phase). Then, the bacteria were diluted serially with LB. The bacteria (including approximately 10^7^ bacterial cells) were exposed to a lethal dose of 50 × MIC of chloramphenicol (Amraei et al., 2020). After incubation 1, 3, and 5 h, mixture was washed three times by PBS to remove the antibiotic. Then the bacteria were diluted 200-folds. The diluents were plated on LB plates and incubated at 28°C for 24 h. Colonies on the plates were counted. The experiment was performed three times.

### Invasion of Host Cell Lines

The interaction of bacteria (TX01, TX01Δ*yoeB*, TX01Δ*yefM-yoeB*, and TX01Δ*yefM-yoeB*C) with Japanese flounder gill cells (FG cells) was performed as below. FG cells were cultured in 96-well cell culture plates to a monolayer and mixed with 100 μL strain (1 × 10^6^ CFU/mL) at a multiplicity of infection (MOI) of 10:1. After incubation at 25°C for 1 and 2 h, the plates were washed three times with PBS. To determine the number of bacterial cells associated with the entire FG cell, the washed FG cells were lysed with 200 μL of 1% (vol/vol) Triton X-100 in PBS, and the number of bacteria was counted by dilution plating (Hu et al., 2014). The experiment was performed three times.

Bacterial replication in murine monocyte-macrophage cells (RAW264.7 cells, cultured at 37°C in 5% CO_2_) was performed as previously (Wang et al., 2019). *E. piscicida* (1 × 10^6^ CFU) was added into RAW264.7 cells cultured in Dulbecco’s minimal Eagle’s medium (DMEM) (Gibco, United States) containing 10% fetal bovine serum (FBS) (Gibco, United States) at a MOI of 10:1. After incubating at 28°C for 2 h, the plates were washed with PBS and extracellular *E. piscicida* was killed by adding gentamicin (100 μg/mL) for 2 h. Then, the cells were washed with PBS and cultured in fresh DMEM containing 10 μg/mL gentamicin for 0, 2, 4, and 6 h. For detecting the number of survival bacteria in RAW264.7 cells at different time point, cells were lysed and lysate were plated on LB agar plates as described above.

### Fish and Experimental Challenges for Bacterial Dissemination *in vivo*

Healthy tilapia (average weight 13.5 g) were purchased from a commercial fish farm of Hainan. The fish were maintained at 25 ± 1°C in aerated water and fed daily with commercial dry pellets. Fish were acclimatized in the laboratory for 2 weeks before experimental manipulation (Fang et al., 2019). To make sure that the fish were not infected by bacteria, randomly selected fish were tested for bacteria in the tissues, including blood, liver, kidney, and spleen. The fish were treated with tricaine methanesulfonate (MS-222) (Sigma, United States) to collect the tissue. For tissue dissemination analysis, TX01, TX01Δ*yoeB*, TX01Δ*yefM-yoeB*, and TX01Δ*yefM-yoeB*C were cultured in LB medium to an OD_600_ of 0.5. The cells were washed with PBS and resuspended in PBS to 10^7^ CFU/mL. Five groups of fish (36 per group) were infected by intramuscularly (i.m.) with 50 μL TX01, TX01Δ*yoeB*, TX01Δ*yefM-yoeB*, TX01Δ*yefM-yoeB*C, and PBS, respectively. At 24 and 48 h post-infection (hpi), spleen from three fish were taken aseptically. The bacterial recovery from the spleen was determined as reported previously (Hu et al., 2014). The rest of the infected fish (30 per group) were used for mortality assay for 25 days. At each examined time points, the result of one experiment was from the average value of three fish. And three replicated experiments were performed.

### Transcriptional Regulation of the Promoter of *yefM-yoeB*

The speculative promoter of *yefM-yoeB* (the 345 bp of upstream of the *yefM-yoeB* operon), P345, was cloned with the primer pair YefM-YoeBP-F/YefM-YoeBP-R and inserted into the SwaI site of pSC11, a promoter probe plasmid (Hu et al., 2009a), which resulted in pSCP345. pSCP345 was introduced into *E. coli* DH5α by transformation and cultured on LB agar plates adding 20 μg/mL X-Gal (5-bromo-4-chloro-3-indolyl-beta-d-galactopyranoside, Solarbio, Beijing, China) (Shi et al., 2019). To construct the overexpression plasmid pTYefM and pTYefM-YoeB, ORFs of *yefM* and *yefM-yoeB* were amplified with the primer pairs YefMF4/YefMR4 and YefMF4/YoeBR4, respectively. The amplified fragments were inserted into the pT3, resulting in pTYefM and pTYefM-YoeB, which express *yefM* and *yefM-yoeB*, respectively. *yefM*, *yefM-yoeB*, and pT (control) were transformed into DH5α/pSCP345, respectively, which resulted in DH5α/pSCP345/pTYefM, DH5α/pSCP345/pTYefM-YoeB, and DH5α/pSCP345/pT. The transformants were cultured on X-gal plates and used for β-galactosidase assay as reported previously (Hu et al., 2009b).

### Expression and Purification of Recombinant YefM (rYefM)

To express the YefM protein, segments of *yefM* were amplified with the primer pair YefMPro-F/YefMPro-R and then the PCR segments were inserted into the protein expression plasmid pET32a, resulting in pET32aYefM. The expressions and purifications of rYefM protein and control protein rTrx were performed as previously (Shi et al., 2019). The Trx (thioredoxin), a tag protein of about 20 KDa in pET32a, can facilitate the soluble expression of exogenous protein.

### Electrophoretic Mobility Shift Assay

An electrophoresis mobility shift assay (EMSA) was performed as below. The DNA fragment of the speculative promoter was amplified by PCR and labeled with carboxyfluorescein (Sangon, China). The labeled DNA was mixed with rYefM and incubated at 37°C for 30 min in 20 μL of binding buffer (1 M Tris–HCl, pH 8.0; 5 M NaCl; 0.1 M MgCl_2_; 0.5 M EDTA; 1 M DTT; 80% glycerol) and the negative control was a mixture of rTrx and nucleic acid probe. The samples were then separated by electrophoresis in non-denaturing 8% polyacrylamide gels (Shi et al., 2019).

### Statistical Analysis

Statistical analyses were performed with SPSS 25.0 software (SPSS Inc., Chicago, IL, United States). Data were analyzed with analysis of variance (ANOVA). Data are expressed as the mean ± standard error of the mean (SEM). Error bars indicate the Standard Error of Mean (SEM) (*n* = 3, biologically independent samples). Statistical significance was defined as *P* < 0.05 (Gu et al., 2021).

## Results

### *E. piscicida* TX01 Harbor YefM-YoeB TA System in Its Genome

**FIGURE 1:**
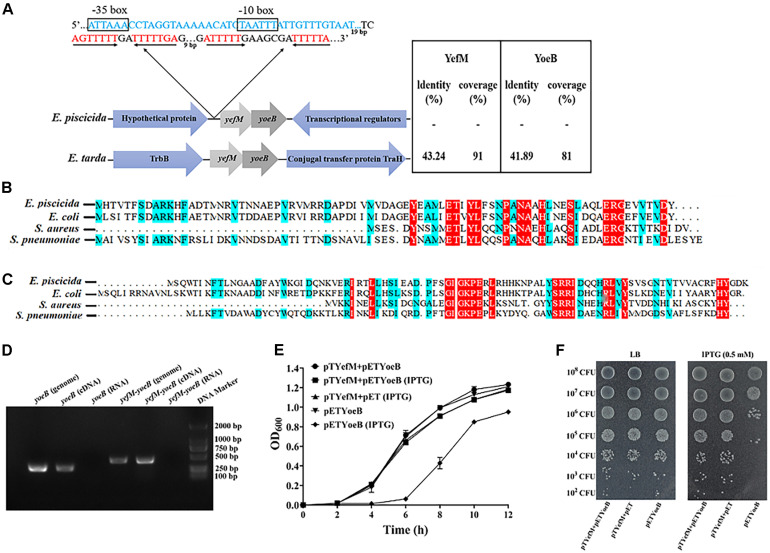
Identification of type II TA system YefM/YeoB in *Edwardsiella piscicida*. **(A)** Genetic organization of YefM/YeoB operon in *Edwardsiella*. The amino acid sequences of *E. piscicida* YefM and YeoB were searched against *Edwardsiella* (taxid:635) database and two bacterial strains of *Edwardsiella* found in the protein BLAST search. Arrows represent genes and genetic context, sizes and spaces are only for representation and are not at actual scale. In the 5′-UTR of *yefM-yoeB*, a promoter region (blue sequence) we predicted and –10 and –35 region were boxed. Two mirror repeat sequences (red sequence) were found in downstream of predicted promoter region. **(B,C)** Sequence alignment of YefM and YoeB from *E. piscicida Escherichia coil*, *Staphylococcus aureus*, and *Streptococcus pneumoniae*, respectively. **(D)** Identification of *yefM* and *yeoB* co-transcription. Total RNA was isolated from *E. piscicida* at a turbidity of 0.8 at 600 nm and treated with DNase I. cDNA was synthesized and used as the template in PCR. PCR products were amplified with specific primer pair YoeBRTF/YoeBRTR for *yoeB* and with primer YefM-YeoBRTF and specific reverse primer YefM-YeoBRTR for *yefM-yeoB*. Genomic DNA was used as a positive control, and total RNA was used as a negative control. **(E)** Inhibition of cell growth by YeoB. Stains BL21/pETYeoB, BL21/pTYefM+Pet, and BL21/pTYefM+pETYeoB were cultured in LB medium with or without IPTG (0.5 mM). **(F)** BL21/pETYeoB, BL21/pTYefM+pET, and BL21/pTYefM+pETYeoB were cultured to the exponential phase, diluted serially and dripped onto the LB plate with or without 0.5 mM IPTG. Data are presented as means ± SEM (*N* = 3). N, the number of times the experiment was performed.

When scaning the whole genome of *E. piscicida* TX01 using TADB 2.0 (Xie et al., 2018), we found Toxin and antitoxin genes. The antitoxin (ETAE_RS07650), a 246 bp ORF, shows homology to the Phd/YefM family. The toxin (ETAE_RS17330), a 276 bp ORF, shows homology to Txe/YoeB family proteins. We named the TA system as YefM-YoeB. Protein BLAST search revealed that only two *Edwardsiella* species, including *E. piscicida*, *E. tarda*, but not *E. ictaluri*, *E. hoshinae*, and *E. anguillarum*, harbor YefM-YoeB TA module and share relatively high homology and similar genetic organization (Figure 1A). Sequence alignment showed that the YefM of *E. piscicida* shares 77.78, 55.26, and 38.75% sequence identities with YefM from *E. coil*, *S. aureus*, and *S. pneumoniae*, respectively (Figure 1B), while YoeB of *E. piscicida* shares 64.04, 44.44, and 45.28% sequence identities with YefM from *E. coil*, *S. aureus*, and *S. pneumoniae*, respectively (Figure 1C).

Classically, two genes of type II TA systems are expressed under one operon, where the antitoxin is located upstream to the toxin (Zander et al., 2020). Consistently, in *E. piscicida*, *yefM* and *yoeB* are adjacent and contain 20 bp overlap sequences. To verify the two genes are co-transcribed, specific primer pair YefM-YoeBRTF/YefM-YoeBRTR that stretch across *yefM* and *yoeB* were designed, and reverse transcription-polymerase chain reaction (RT-PCR) was conducted using total RNA, complementary DNA (cDNA), and genomic DNA (gDNA) of *E. piscicida* as templates. The results showed that the predicted PCR products of 447 bp were observed in cDNA and gDNA templates, but not in RNA template (Figure 1D), which indicates that *yefM* and *yoeB* are expressed in a bicistronic operon.

When TA system is working, the toxin protein exhibits its inhibitory effect on the growth of the bacteria, while the antitoxin protein eliminates the toxic effect (Chan et al., 2012). To check the interaction between YefM and YoeB, pTYefM, which expresses antitoxin YefM constitutively, and pETYoeB, which expresses YoeB inductively by IPTG, were constructed and transformed into BL21, respectively, or simultaneously. The growth curve assay was performed and the result showed that the growth of BL21/pETYoeB was similar to that of BL21/pTYefM+pETYoeB. However, when IPTG was added in the medium, the growth of BL21/pETYoeB was delayed markedly, but the growth of BL21/pTYefM+pETYoeB did not affected by IPTG (Figures 1E,F). This result indicates that YoeB has a toxic effect on BL21 cells, and the effect was neutralized by YefM. These findings show that YefM-YoeB is a characteristic type II TA system.

### Construction of yoeB and *yefM-yoeB* Mutants

To examine their functional importance, the *yoeB* and *yefM-yoeB* were knocked out by markerless in-frame deletion of the operon from 37 to 198 bp and 67 to 469 bp, respectively. The resulting mutants were named TX01Δ*yoeB* and TX01Δ*yefM-yoeB*, respectively. However, we did not obtain the *yefM* mutant, which indicates the absence of YefM-type antitoxins seems to be lethal in different bacteria or at least to affect their growth (Yoshizumi et al., 2009). Next, we examined the effects of TX01Δ*yoeB* and TX01Δ*yefM-yoeB* on the information of persister cells, adversity adaptation, and pathogenicity of *E. piscicida*.

### Effects of yoeB and yefM-yoeB Mutants on the Persister Cells Populations

**FIGURE 2:**
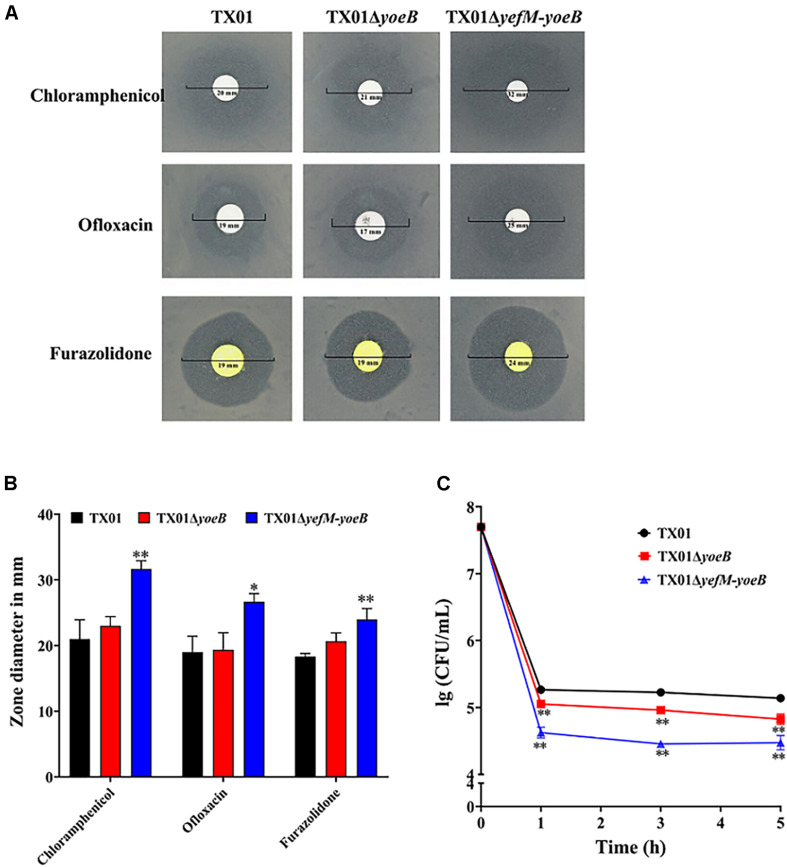
Resistance of *Edwardsiella piscicida* to antibiotics and their ability to form persistent bacteria. **(A)** Drug sensitivity test of *E. piscicida*. Thirty drug sensitive papers were selected to determine the drug resistance of TX01, TX01Δ*yoeB*, TX01Δ*yefM-yoeB* by Kirby-Bauer. The three bacteria were sensitive to chloramphenicol, ofloxacin, and furazolidone, among which chloramphenicol was the most sensitive antibiotics. **(B)** The inhibition zone of chloramphenicol, ofloxacin, and furazolidone on TX01, TX01Δ*yoeB*, and TX01Δ*yefM-yoeB*. **(C)** Time-kill curves of different strains exposed to chloramphenicol. TX01, TX01Δ*yoeB*, TX01Δ*yefM-yoeB* were exposed to lethal concentration (240 μg/mL) of chloramphenicol for 1, 3, and 5 h, then viable bacteria were determined by culturing on the LB agar plates. Data are presented as the means ± SEMs (*N* = 3). N, the number of times the experiment was performed. ^∗^*P* < 0.05; ^∗∗^*P* < 0.01.

Studies have proved that the majority of type II TA systems are related to the formation of persistence bacteria (Amraei et al., 2020), so we want to investigate the participation of YefM-YoeB in persister cells of *E. piscicida*. By means of the drug susceptibility test, we found that TX01, TX01Δ*yoeB*, TX01Δ*yefM-yoeB* were sensitive to chloramphenicol, ofloxacin, and furazolidone, among which chloramphenicol was the most sensitive antibiotic. Amongst the three strains, TX01Δ*yefM-yoeB* was more sensitive to the all three antibiotics than TX01Δ*yoeB*, which was comparative to TX01 (Figures 2A,B). Based on this result, we speculated that YefM-YoeB play a role in the resistance of bacteria against the three antibiotics. Chloramphenicol was chosen for the test of bacterial persistence formation. Then the minimum inhibitory concentration (MIC) assay was conducted and the result showed that the MIC of chloramphenicol on TX01, TX01Δ*yoeB*, TX01Δ*yefM-yoeB* were all 4.8 μg/mL. Then the lethal concentration 240 μg/mL (50 × MIC) of chloramphenicol was used to treat these strains. Viable bacteria were determined and the result showed the number of persistence bacteria of TX01Δ*yoeB* and TX01Δ*yefM-yoeB* were significantly reduced by about twofolds and fivefolds compared to TX01, respectively (Figure 2C), indicating *yoeB* and *yefM-yoeB* play an important role in the formation of persister cell of *E. piscicida*.

### Effects of *yoeB* and *yefM-yoeB* Mutants on Bacterial Resistance Against Environmental Stress

**FIGURE 3:**
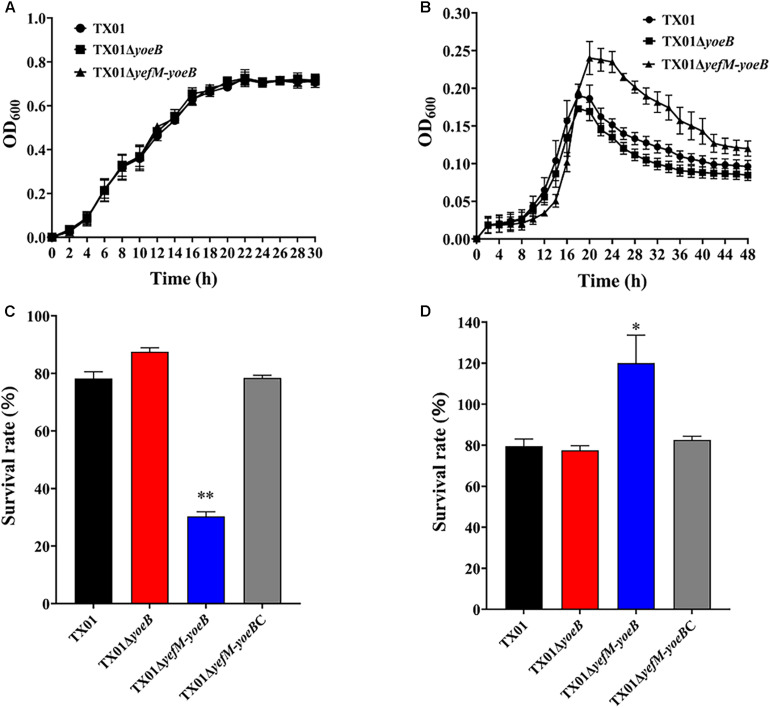
Sensitivity of *Edwardsiella piscicida* to stress. **(A)** The growth curve of TX01, TX01Δ*yeoB*, and TX01Δ*yefMyeoB* in LB medium under normal condition. **(B)** The growth curve of TX01, TX01Δ*yeoB*, and TX01Δ*yefMyeoB* in LB medium under high temperature (40°C). **(C)** The survival rate of TX01, TX01Δ*yeoB*, TX01Δ*yefMyeoB*, and TX01Δ*yefMyeoB*C under oxidation stress. Strains were cultured to logarithmic phase in normal LB medium, then bacteria were incubated with H_2_O_2_ (3.2 mM) or PBS for 1 h. The number of the viable bacteria was determined by plate counting. **(D)** The survival rate of TX01, TX01Δ*yeoB*, TX01Δ*yefMyeoB*, and TX01Δ*yefMyeoB*C against non-immune fish serum. Strains in the early logarithmic phase were incubated with non-immune tilapia serum or PBS (control) for 1 h. The number of the viable bacteria was determined by plate counting. Data are the means of three independent experiments and presented as means of three independent experiments and presented as means ± SEM (*N* = 3). N, the number of times the experiments was performed. ^∗^*P* < 0.05; ^∗∗^*P* < 0.01.

One of the functions of TA systems is to help bacteria adapt to adversity. High temperature is a kind of environmental pressure that bacteria often undergo. So we firstly examined the effect of YefM-YoeB on bacterial resistance against temperature stress. For this purpose, growth analysis was performed and the result showed that TX01, TX01Δ*yoeB*, and TX01Δ*yefM-yoeB* exhibited a comparative growth rate when bacteria were cultured in LB medium under normal condition (Figure 3A), which indicates deletion of *yoeB* and *yefM-yoeB* did not affect bacterial growth and propagation in normal condition. However, when cultured in LB medium at high temperature (40°C), TX01Δ*yefM-yoeB* displayed higher maximum cell density than TX01, while TX01Δ*yoeB* exhibited growth patterns similar to that of TX01 (Figure 3B). These results suggest that the deletion of *yoeB* did not affect bacterial tolerance to high temperature, but the deficiency of the whole TA system *yefM-yoeB* enhanced bacterial tolerance to high temperature stress.

Oxidative stress and acid stress are unavoidable challenge for pathogenic bacteria invading and living in the host. To check the role of this TA system in oxidative stress, TX01, TX01Δ*yoeB*, and TX01Δ*yefM-yoeB* at logarithmic phase were transferred to new LB medium with or without oxidative stress (H_2_O_2_) and cultured for another 1 h, then the number of the viable bacteria was determined by plate counting. The result showed that under the condition of oxidative pressure, the survival amount of TX01Δ*yoeB* was comparative to that of TX01, but the survival amount of TX01Δ*yefM-yoeB* was significantly lower than that of TX01 (Figure 3C). The result indicates that the deletion of the whole TA system *yefM-yoeB* impairs the ability of *E. piscicida* to resist oxidative stress, but only the deficiency of *yoeB* had no effect on bacterial resistance against oxidative stress. To examine the role of the TA system in acid pressure, strains at logarithmic phase was transferred to new normal LB medium (pH = 7.0) or acidic LB medium (pH = 4) and cultured for another 1 h, then the number of the viable bacteria was determined. The result showed that survival rate of three strains (TX01, TX01*yoeB*, and TX01Δ*yefM-yoeB*) did not displayed significantly difference (data not shown), which indicates YefM-YoeB TA system is not related to acid tolerance.

### Effects of *yoeB* and *yefM-yoeB* Mutants on Bacterial Resistance Against Non-immune Fish Serum

Host serum bactericidal activity is a vital immune weapon for eliminating pathogens. Resistance against host serum killing is an important characteristic of *E. piscicida* (Li et al., 2015). To examine whether *yoeB* and *yefM-yoeB* mutation affect the ability of *E. piscicida* to tolerate host serum stress, TX01, TX01Δ*yoeB*, and TX01Δ*yefM-yoeB* were mixed with tilapia serum, and viable bacteria was detected by plat counting. As shown in Figure 3D, TX01 and TX01Δ*yoeB* exhibited similar capability of serum resistance, as 79 and 77% of bacteria survived after treatment with tilapia serum, respectively. In contrast, compared to TX01 and TX01Δ*yoeB*, TX01Δ*yefM-yoeB* demonstrated significantly stronger resistance ability to host serum, as 120% of cells survived. The result showed TA system YefM-YoeB of *E. piscicida* participated in the host serum resistance, indicating the probable involvement of YefM-YoeB in pathogenicity of *E. piscicida*. As far as we known, this is the first study about the relationship between type II TA system and serum resistance.

### Effects of *yoeB* and *yefM-yoeB* Mutants on Bacterial Biofilm Formation

**FIGURE 4:**
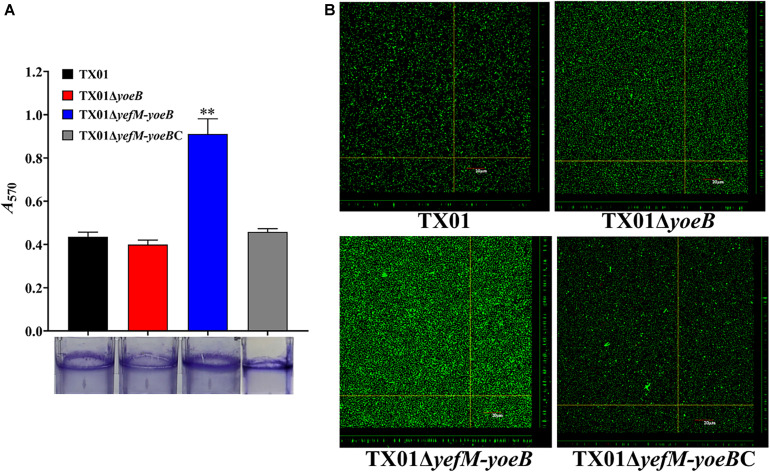
Effects of *yeoB* and *yefMyeoB* mutation on bacteria biofilm growth. **(A)** Biofilm-forming capacity of *E. piscicida*. TX01, TX01Δ*yeoB*, TX01Δ*yefMyeoB*, and TX01Δ*yefMyeoB*C were incubated in polystyrene plates, and biofilm formation was determined by measuring the *A*_570_ of the final eluates of crystal violet staining. Data are presented as the means ± SEMs (*N* = 3). N, the number of times the experiment was performed. ^∗∗^*P* < 0.01. **(B)** The viability of biofilm growth of *E. piscicida* as determined by confocal laser scanning microscopy (CLSM). Cells in the biofilms were stained with a BacLight LIVE/DEAD kit to reveal viable (green fluorescence) and non-viable (red fluorescence) bacteria.

Bacterial biofilm formation is also closely related with pathogenicity. Since type II TA systems contribute extensively to bacterial biofilm (Fraikin et al., 2019), we investigated the relationship between YefM-YoeB and biofilm growth of *E. piscicida*. For this purpose, TX01, TX01Δ*yoeB*, and TX01Δ*yefM-yoeB* were cultured in polystyrene plate. The biofilm formed around the plate was fixed with Bouin and dyed with crystal violet. The subsequent result indicated that the biofilm formation of TX01Δ*yefM-yoeB* was remarkably higher than those of TX01 and TX01Δ*yoeB*, the latter two exhibited similar biofilm forming ability (Figure 4A). To confirm the result, we acquired images of the biofilms using CLSM. The result displayed that compared to wild type TX01, TX01Δ*yefM-yoeB* exhibited a substantial increase in the thickness and density of the biofilm, and TX01Δ*yoeB* presented a similar level to TX01 (Figure 4B). These results suggest that the deletion of TA system YefM-YoeB increases the ability of *E. piscicida* to form biofilm, indicating the probable involvement of YefM-YoeB in pathogenicity of *E. piscicida*.

### Effects of *yoeB* and *yefM-yoeB* Mutants on Cell Invasion and Intracellular Survival

**FIGURE 5:**
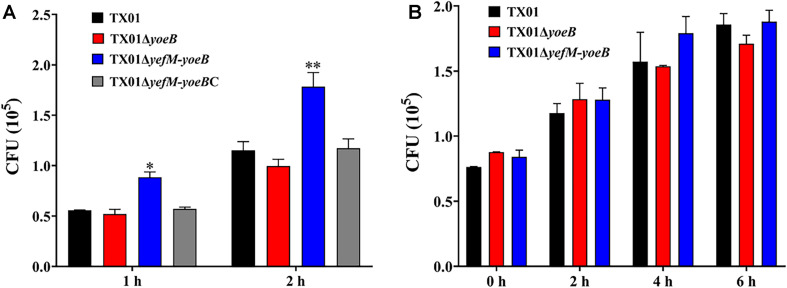
Effects of *yeoB* and *yefMyeoB* mutation on cellular infection and replication. **(A)** The association and invasion of *E. piscicida* to flounder gill cells (FG cells). FG cells were infected with the same dose of TX01, TX01Δ*yeoB*, TX01Δ*yefMyeoB*, and TX01Δ*yefMyeoB*C for various time and washed with PBS. Then the FG cells were lysed, the bacteria associated with and invaded into the host cells were determined by plate counting. **(B)** Survival and replication of *E. piscicida* in mouse macrophage cell RAW264.7. RAW264.7 cells were hatched with TX01, TX01Δ*yeoB*, TX01Δ*yefMyeoB* for 2 h, then the mouse macrophages were treated with gentamicin for 2 h. After the cells were incubated with bacterial for various hours, the number of intracellular bacteria was determined by plate counting. Data are presented as the means ± SEMs (*N* = 3). N, the number of times the experiment was performed. ^∗^*P* < 0.05; ^∗∗^*P* < 0.01.

Since the deletion of *yefM-yoeB* has significant impact on serum resistance and biofilm information, we infer that TA system YefM-YoeB is involved in the virulence of *E. piscicida*. To examine the role of YefM-YoeB in the infectivity of *E. piscicida*, cultured FG cells were infected with the same number of TX01, TX01Δ*yoeB*, and TX01Δ*yefM-yoeB* for 1 and 2 h. After washing with PBS, the bacterial that were adhered with and invaded into the host cells were detected. As shown in Figure 5A, the amount of TX01Δ*yefM-yoeB* obtained from FG cells was markedly higher than that of wild strain at 1 h and 2 hpi, however, as we expected, the amount of TX01Δ*yoeB* was comparative to that of TX01 (Figure 5A). The result shows that the deficiency of *yefM-yoeB*, but not *YoeB*, significantly enhanced the invasion of *E. piscicida* to host cells, which is consistent with results of serum resistance and biofilm information.

Survival and replication in host macrophages is a typical characteristic of *E. piscicida* (Sui et al., 2017). We want to examined the participation of YefM-YoeB in intracellular survival of phagocyte. For this purpose, RAW264.7 cells were infected with the same number of three *E. piscicida* strains. After killing the extracellular bacteria, the cells were cultured further in normal medium for different time, then the amount of intracellular bacteria was detected. Unexpectedly, the results displayed that the bacterial number of TX01Δ*yefM-yoeB* from the intracellular RAW264.7 cells was comparative to those of TX01 and TX01Δ*yoeB* (Figure 5B), which indicates that type II TA system YefM-YoeB is not involved in the intracellular survival of macrophage.

### Effects of *yoeB* and *yefM-yoeB* Mutants on Bacterial Dissemination in Tissues and General Virulence

**FIGURE 6:**
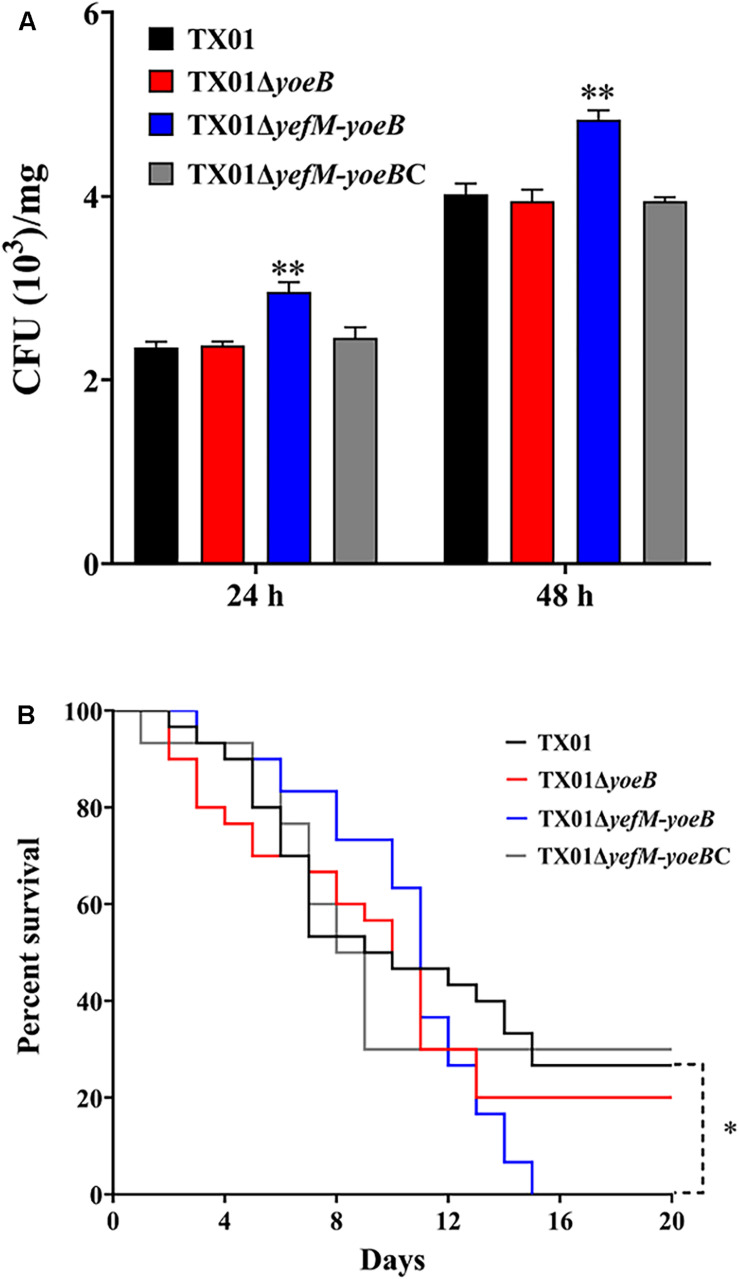
Effects of *yeoB* and *yefMyeoB* mutation on bacterial dissemination of fish tissues and general virulence. **(A)** Bacterial dissemination in the fish tissues. Tilapia were infected with the same dose of TX01, TX01Δ*yeoB*, TX01Δ*yefMyeoB*, and TX01Δ*yefMyeoB*C, and bacterial recovery from the spleen was determined by plate counting at 48 and 72 h post-infection. **(B)** Host mortality induced by *E. piscicida*. Tilapia were infected with equivalent doses of TX01, TX01Δ*yeoB*, TX01Δ*yefMyeoB*, and TX01Δ*yefMyeoB*C, and accumulated mortality were monitored for a period of 25 days (only 20 days are shown since no more deaths occurred after 20 days). Significance between the survivals of wild type and mutant infected fish was determined with logrank test. Data are presented as the means ± SEMs (*N* = 3). N, the number of times the experiment was performed. ^∗^*P* < 0.05; ^∗∗^*P* < 0.01.

To further clarify the participation of the TA system in the virulence of *E. piscicida*, *in vivo* experiment of tissue infectivity was performed. The same dose of TX01, TX01Δ*yoeB*, and TX01Δ*yefM-yoeB* were used to infect Tilapia and viable bacteria from spleen were determined after infection 24 and 48 h. As shown in Figure 6A, bacteria amount from TX01Δ*yefM-yoeB*-infected fish was significantly higher than that of TX01-infected fish, but TX01Δ*yoeB*-infected fish was comparative to that of TX01-infected fish, which is consistent with the result of invading FG cells.

To detect the effect of *yefM-yoeB* mutation on bacterial virulence, fish were infected with TX01, TX01Δ*yoeB*, and TX01Δ*yefM-yoeB* and the mortality of fish was monitored. The results displayed that at the end of the monitored period (25 days), the survival rate of TX01- and TX01Δ*yoeB*-infected fish was 29 and 20%, respectively, which was significantly higher than that of TX01Δ*yefM-yoeB*-infected fish (0%) (Figure 6B). These findings indicate the deletion of the whole TA system enhances the pathogenicity of *E. piscicida*.

### Genetic Complementation of the *yefM-yoeB* Deletion and Its Effect on Stress Resistance and Virulence

To further clarify the function of *yefM-yoeB*, the complementary strain of *yefM-yoeB* mutant TX01Δ*yefM-yoeB*, TX01Δ*yefM-yoeB*C, was constructed. In contrast to TX01Δ*yefM-yoeB*, TX01Δ*yefM-yoeB*C exhibited a comparative resistance against oxidative stress and host serum killing and a comparative capacity of biofilm growth to those of TX01 (Figures 3, 4). Likewise, the bacterial infective and dissemination capacities of TX01Δ*yefM-yoeB*C in host cells and tissues were comparable to those of TX01 (Figures 5, 6).

### The Expression of *yefM-yoeB* Was Regulated by YefM and YoeB

**FIGURE 7:**
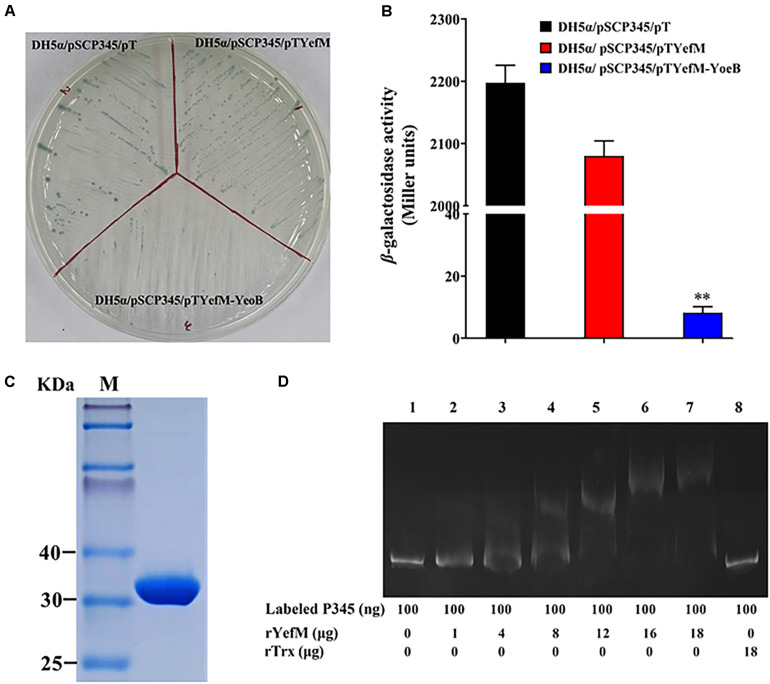
Expression of *yefMyeoB* was regulated by YefM and YefM/YeoB. **(A)** DH5α/pSCP345/pT, DH5α/pSCP345/pTYefM, and DH5α/pSCP345/pTYefM-YeoB were streaked and cultured on LB plates with X-gal, kanamycin, and ampicillin. **(B)** The β-galactosidase activities of DH5α/pSCP345/pT, DH5α/pSCP345/pTYefM, and DH5α/pSCP345/pTYefM-YeoB was determined and shown by Miller units. Data are presented as the means ± SEMs (*N* = 3). N, the number of times the experiment was performed. ^∗∗^*P* < 0.01. **(C)** Purified rYefM was analyzed by SDS-PAGE. **(D)** Interaction between YefM and the speculative promoter regions of *yefMyeoB*. An electrophoresis mobility shift assay (EMSA) was performed in binding buffer containing rYefM, rTrx (control), carboxyfuorescein-labeled P345, and the speculative promoter regions (P345) of *yefMyeoB*. The negative control DNA.

In most type II TA systems, the antitoxin and toxin proteins participate in transcriptional autoregulation under stress conditions (Kedzierska et al., 2007; Overgaard et al., 2008). In the 5′-UTR of *yefM-yoeB*, a promoter region was predicted and two mirror repeat sequences were found (Figure 1A). To survey the regulatory effect of YefM-YoeB on its own expression, the speculative promoter of *yefM-yoeB*, P345, was inserted into pSC11, the recombinant strain was named DH5α/pSCP345. When DH5α/pSCP345 was grew on LB agar plates including X-gal, the bacterial colonies were blue, which indicates that P345 possesses promoter activity. Then pTYefM (expressing YefM), pTYefM-YoeB (expressing YefM-YoeB) and control plasmid pT were transformed into DH5α/pSCP345, respectively. On the same X-gal plate, the blue of DH5α/pSCP345/pTYefM was slightly weaker than that of DH5α/pSCP345/pT, and DH5α/pSCP345/pTYefM-YoeB was obviously weaker than that of DH5α/pSCP345/pTYefM (Figure 7A). β-galactosidase assay displayed that the Miller units produced by DH5α/pSCP345/pTYefM were moderately lower than that produced by DH5α/pSCP345/pT, and the Miller units produced by DH5α/pSCP345/pTYefM-YoeB was significantly lower than that produced by DH5α/pSCP345/pT (Figure 7B). These results suggested that YefM-YoeB negatively regulated the expression of *yefM-yoeB* transcription. Next, we wanted to explore the interaction between YefM-YoeB and the promoter of *yefM-yoeB*. We did not obtain the complex of rYefM and rYoeB, but rYefM was successfully expressed and purified from *E. coli* (Figure 7C). EMSA was performed and the result showed that purified rYefM could bind the P345 (Figure 7D), which suggests that YefM-YoeB could regulate directly the expression of operon *yefM-yoeB*.

## Discussion

In the course of evolution, pathogens usually assimilate and integrated exogenous genetic materials into its genome to develop quick adaptive advantages in intricate habitat. As a product of evolution, type II TA system are often found in chromosomes and some mobile components such as plasmids and prophages (Harms et al., 2018). Recently, type II TA system has attracted increasing attention since its important roles in physiology, stress, and pathogenicity (Wang and Wood, 2011; Bukowski et al., 2013; Harms et al., 2018; Równicki et al., 2020). As a leading pathogen threatening worldwide aquaculture industries, *E. piscicida* has received more and more attention and has been considered as a model organism for studying intracellular and systemic infections (Leung et al., 2019). However, for all we know, TA systems are totally unknown in *E. piscicida* so fa. By means of TADB 2.0 (Xie et al., 2018), we performed a scan for whole genome of *E. piscicida* TX01 and found the YefM and YoeB. Bioinformatics analysis showed that YefM and YoeB share high homology with type II toxin-antitoxin system Phd/YefM family antitoxin and toxin YoeB, respectively. *yefM* and *yoeB* are transcriptionally organized in a bicistronic operon, and *yefM* locates upstream of the *yoeB*, which is one of typical characteristic of most of type II TA system (Yamaguchi et al., 2011). We name the bicistronic operon as type II TA system YefM-YoeB. It has been reported that the function of the toxin molecule is often associated with the inhibition of DNA replication or protein translation, resulting in cell growth arrest, which was neutralized by its cognate antitoxin protein (Page and Peti, 2016; Chan et al., 2018; Równicki et al., 2020). Consistently with other type II TA system, we found when YoeB of *E. piscicida* expressed in *E. coli*, the growth of strain delayed obviously, and the toxic effect of YoeB was relieved by YefM. These results indicate that YefM-YoeB is a functional type II TA system of *E. piscicida*.

The emergence of antibiotic-resistant bacteria is a tricky and confronted issue in modern medicine, and one of important reasons is the widespread of toxin-antitoxin (TA) systems in pathogenic bacteria (Równicki et al., 2020). The TA system usually induces cells to enter a dormant state so that the cells can escape the effects of antibiotics (Wang et al., 2011). As reported in *E. coli*, the number of persistent cells of *E. coli* lacking *mqsRA* is significantly reduced under high concentration of antibiotics (Kim and Wood, 2010). Compared with the wild type, the cell survival rate of *E. coli* lacking *yafQ* under the bactericidal concentration of antibiotics decreased by 2,400-folds (Harrison et al., 2009). Studies have shown that in *E. coli*, the MazEF plays an important role in the formation of persistent bacteria and the MazF-mediated persistence phenotype was found to dependent on the presence of the ClpP and Lon proteases (Tripathi et al., 2014). In this study, we found the persister forming ability of TX01Δ*yoeB* and TX01Δ*yefM-yoeB* was significantly reduced compared to that of TX01, which indicates the involvement of YefM-YoeB in bacterial persister formation, but its mechanism needs further study.

Response to environmental stress is one of prominent functions of TA systems. Under pressure, the labile antitoxins are proteolytically degraded, which releases toxins. Exposed toxins inhibit bacterial growth and help bacteria survival in adversity (Christensen-Dalsgaard et al., 2010; Bukowski et al., 2013). For survival in host, pathogen must overcome the oxidative stress (Forman and Torres, 2002). It is reported that multiple type II TA system genes in *Klebsiella pneumoniae* were involved in response to oxidative stress (Narimisa et al., 2020). In *E. coli*, upon oxidative stress, antitoxin MqsA is degraded and toxin MqsR was liberated, which enriched ghoT mRNAs as well as other stress-related transcripts and leaded to the formation of persister cells (Wang et al., 2013). However, the deletion of whole TA system MqsRA have no effect on oxidative stress resistance (Fraikin et al., 2019). On the contrary, the deletion of whole TA system YefM-YoeB in *S. pneumoniae* leads to reduced resistance to oxidative stress (Chan et al., 2018). Consistently with the result in *S. pneumoniae*, we found the deficiency of the whole TA system *yefM-yoeB* impaired the ability of *E. piscicida* to resist oxidative stress, but only the inactivation of *yoeB* had no effect on resistance against oxidative stress, which indicates YefM perhaps plays an important role in oxidative pressure reaction, since deletion of *yoeB* releases YefM. In contrast to the result of oxidative stress, the deletion of whole TA system YefM-YoeB leads to enhance resistance of *E. piscicida* to high temperature stress and host serum stress. These observations in *E. coli* suggest that YefM-YoeB functions in adaptation to temperature stress (Janssen et al., 2015). As far as we known, there is no report of YefM-YoeB about serum resistance. All in all, these results suggest YefM-YoeB are extensively involved in response to stress and its functions in different pathogens are not in complete accord.

Accumulating reports have confirmed that TAs are involved in multiple important biological processes, such as biofilm formation (Eroshenko et al., 2020). For example, in the MazEF type II TA pair, overexpression of *mazF* toxin drives to the inhibition of biofilm formation (Ma et al., 2019). In HigBA pair, *higA* mutant and *higB* overexpression strain reduced the biofilm formation, which indicates HigB represses biofilm formation of *P. aeruginosa* (Wood and Wood, 2016). In the MqsR-MqsA pair, MqsA decreases the production of CsgD, the master regulator for biofilm formation (Soo and Wood, 2013). The deficiency of *yefM-yoeB* operon in *S. pneumoniae* bring about a considerable decrease in the thickness of the biofilm (Chan et al., 2018). However, in this study, deletion of *YoeB* did not affect the biofilm growth, but deletion of *yefM-yoeB* operon exhibited an increase of biofilm formation, which indicated the participation of YefM-YoeB TA system in the biofilm of *E. piscicida*. However, the role of YefM-YoeB in biofilm formation in *E. piscicida* are contrary to the role in *S. pneumoniae* (Chan et al., 2018), which suggests the function and mechanism of YefM-YoeB in different pathogen are different, and also urges us to study and reveal these TA systems.

Increasing evidences confirm that TA systems have emerged as important elements that affect the pathogenicity of many bacteria (Wang and Wood, 2011; Harms et al., 2018; Równicki et al., 2020). For example, antitoxin HigA inhibits virulence gene *mvfR* expression in *P. aeruginosa* (Guo et al., 2019). The TA system SehA/SehB in *Salmonella* promotes bacterial infection in mice (De la Cruz et al., 2013). Since the inactivation of *yefM-yoeB* affects the resistance against adversity stress, biofilm growth, and host serum killing, which are related to the virulence (Shi et al., 2019; Wang et al., 2019; Jiang et al., 2020), so we examined role of YefM-YoeB in pathogenicity of *E. piscicida*. As we expected, *in vitro* experiment demonstrated that the deficiency of *yefM-yoeB* significantly increased the capacity of *E. piscicida* to adhere and invade to host cells. Consistently, *in vivo* experiment showed that the deletion of *yefM-yoeB* significantly enhanced the ability of to disseminate in the fish tissues and elevated general virulence. Introduction of an in trans-expressed *yefM-yoeB* restored the virulence of TX01Δ*yefM-yoeB*. In contrast to our results, deletion of the *yefM-yoeB* locus had no effect on the virulence of *Streptococcus suis* (Zheng et al., 2015). However, we found that only deletion of the *yoeB* also had no effect on the virulence of *E. piscicida*. Moreover, TX01Δ*yefM-yoeB* and TX01 exhibited the similar ability of survival and replication in macrophages. Taking all the above results together, we believe that YefM-YoeB participates the virulence of *E. piscicida*, but its mechanism is complex and different from other pathogens. In *E. piscicida*, we found *TX01ΔyefM-yoeB* displayed obvious difference with *TX01ΔyoeB* in stress resistance and pathogenicity. We speculate when *yoeB* is deleted, another toxin protein that is functionally and structurally similar to YeoB could bind with YefM, just as the finding that YeeU is structurally similar to toxins YoeB in *E. coli* (Arbing et al., 2010). Therefor the phenotype of *TX01ΔyoeB* was similar to that of *TX01* but different from that of *TX01ΔyefM-yoeB*, which is totally missing the TA system.

In typical type II TA systems, the antitoxin alone or the TA complex binds to the promoter and regulates self the transcription of the TA operon (Page and Peti, 2016). In type II TA systems of *S. suis*, RelB1, RelB2, ParD, and ParDE negatively autoregulated the transcriptions of their respective TA operons, while RelBE2 positively autoregulated its TA operon transcription (Xu et al., 2018). In *Streptomyces lividans*, YefM-YoeB complex purified from *E. coli* could bind with high affinity to its own promoter region (Sevillano et al., 2012). Similarly, in *E. piscicida*, we also found YefM-YoeB dramatically inhibited the activity of the promoter of *yefM-yoeB*. Moreover, we found YefM directly bound with its own promoter. These results indicate that YefM-YoeB functions as transcriptional regulator and negatively autoregulate the its own expression.

In conclusion, we identified type II TA system YefM-YoeB in *E. piscicida*, an important pathogen threatening worldwide aquaculture industries. Our results show that the deletion of *yefM-yoeB* impaired the ability of *E. piscicida* to oxidative resistance and reduced the persistence bacteria formation ability, but enhanced the ability of biofilm formation, serum resistance, and host infection. The inactivation of yoeB has no effects on resistance to stress and virulence. YefM-YoeB negatively autoregulated its own expression and YefM can directly bind to the promoter of *yefM-yoeB*. This study provides new insights into the biological activity of type II TA system YefM-YoeB in aquatic pathogenic bacteria.

## Data Availability Statement

The original contributions presented in the study are included in the article/supplementary material, further inquiries can be directed to the corresponding author/s.

## Ethics Statement

The animal study was reviewed and approved by the ethics committee of Institute of Tropical Bioscience and Biotechnology, Chinese Academy of Tropical Agricultural Sciences. Efforts were taken to ensure that all research animals received good care and humane treatment.

## Author Contributions

DMM carried out the experiments. HJG analyzed the data and revised the manuscript. YJS participated in gene knock-out experiments. HQH participated in the infection experiments. DMS participated in the experiment design and drafted the manuscript. HYH designed the experiments, interpreted results, and wrote the manuscript. All authors contributed to the article and approved the submitted version.

## Conflict of Interest

The authors declare that the research was conducted in the absence of any commercial or financial relationships that could be construed as a potential conflict of interest.
